# Reactivation of Estrogen Receptor α by Vorinostat Sensitizes Mesenchymal-Like Triple-Negative Breast Cancer to Aminoflavone, a Ligand of the Aryl Hydrocarbon Receptor

**DOI:** 10.1371/journal.pone.0074525

**Published:** 2013-09-13

**Authors:** Karri Stark, Angelika Burger, Jianmei Wu, Phillip Shelton, Lisa Polin, Jing Li

**Affiliations:** 1 Karmanos Cancer Institute, Wayne State University, Detroit, Michigan, United States of America; 2 Department of Pharmacology, University of Maryland, Baltimore, Maryland, United States of America; University of Chicago, United States of America

## Abstract

**Objective:**

Aminoflavone (AF) acts as a ligand of the aryl hydrocarbon receptor (AhR). Expression of estrogen receptor α (ERα) and AhR-mediated transcriptional induction of CYP1A1 can sensitize breast cancer cells to AF. The objective of this study was to investigate the combined antitumor effect of AF and the histone deacetylase inhibitor vorinostat for treating mesenchymal-like triple-negative breast cancer (TNBC) as well as the underlying mechanisms of such treatment.

**Methods:**

*In vitro* antiproliferative activity of AFP464 (AF prodrug) in breast cancer cell lines was evaluated by MTS assay. *In vitro,* the combined effect of AFP464 and vorinostat on cell proliferation was assessed by the Chou-Talalay method. *In vivo,* antitumor activity of AFP464, given alone and in combination with vorinostat, was studied using TNBC xenograft models. Knockdown of ERα was performed using specific, small-interfering RNA. Western blot, quantitative RT-PCR, immunofluorescence, and immunohistochemical staining were performed to study the mechanisms underlying the combined antitumor effect.

**Results:**

Luminal and basal A subtype breast cancer cell lines were sensitive to AFP464, whereas basal B subtype or mesenchymal-like TNBC cells were resistant. Vorinostat sensitized mesenchymal-like TNBC MDA-MB-231 and Hs578T cells to AFP464. It also potentiated the antitumor activity of AFP464 in a xenograft model using MDA-MB-231 cells. *In vitro* and *in vivo* mechanistic studies suggested that vorinostat reactivated ERα expression and restored AhR-mediated transcriptional induction of *CYP1A1*.

**Conclusion:**

The response of breast cancer cells to AF or AFP464 was associated with their gene expression profile. Vorinostat sensitized mesenchymal-like TNBC to AF, at least in part, by reactivating ERα expression and restoring the responsiveness of AhR to AF.

## Introduction

AFP464, a lysine prodrug of aminoflavone (AF; NSC 686288), is a novel anticancer drug that is currently in clinical investigational trials to treat breast cancer. AFP464 was synthesized to improve the solubility of the parent compound. It undergoes rapid conversion to AF by nonspecific esterases in plasma and cell culture medium. Mechanistic studies of the cytotoxicity of AF have shown that AF induces DNA damage associated with DNA-protein cross-linking, DNA single-strand breaks, and DNA replication-dependent double-strand breaks [1], [2]. AF produces a unique COMPARE pattern of activity in the panel of 60 NCI cell lines. This unique pattern of tumor sensitivity has been attributed to the need for intracellular bioactivation by cytochrome P450 1A1 (CYP1A1) and sulfotransferase 1A1 (SULT1A1) to convert AF to DNA-damaging species [3]–[6]. Notably, AF can induce its own metabolism by activating transcription of *CYP1A1* through the aryl hydrocarbon receptor (AhR) pathway [3], [4].

The AhR is a ligand-activated transcription factor that binds a wide range of endogenous and xenobiotic compounds [7]. In the absence of ligand, the AhR is bound to a multi-chaperone protein complex located in the cytoplasm [8]. Upon ligand binding, the AhR translocates to the nucleus where it binds to its dimerization partner, the aryl hydrocarbon nuclear translocator (ARNT). Subsequently, the activated AhR/ARNT heterodimer binds to its cognate DNA sequences (termed xenobiotic response elements) and recruits coregulators, leading to transcriptional activation of AhR target genes, including but not limited to *CYP1A1* [9], [10]. AF acts as a ligand of the AhR. Data from our laboratory [11] and other groups [4] suggest that the sensitivity of cancer cells to AF is associated with cytoplasmic expression of AhR. In AF-sensitive cancer cells, the AhR is present in the cytoplasm as a component of a complex with the chaperone heat shock protein (Hsp90). Acting as a ligand of the AhR, AF binds to the cytoplasmic AhR, causing it to translocate to the nucleus which subsequently leads to transcriptional activation of AhR target genes, including but not limited to *CYP1A1*, that convert AF to DNA-damaging species [4]. Conversely, in AF-resistant cancer cells, the AhR is localized in the nucleus, and AF cannot activate the AhR pathway [4]. Although constitutive expression of *SULT1A1* has been associated with cancer cell sensitivity to AF [6], AhR’s responsiveness to AF, as indicated by induction of *CYP1A1*, appears to be essential for the antiproliferative activity of AF in breast cancer cell lines [3], [4].

AF or AFP464 exhibits differential *in vitro* antiproliferative activity in human breast cancer cell lines. Notably, estrogen receptor (ER)-positive breast cancer cell lines, irrespective of resistance to anti-estrogen or anti-HER2 therapies (e.g., tamoxifen refractory MCF-7/TAM1 and herceptin refractory MCF-7/Her2-18 cell lines), were sensitive to AF, whereas triple-negative breast cancer cell (TNBC) lines with the molecular characteristics of basal B or mesenchymal-like subtypes (e.g., MDA-MB-231 and Hs578T) [12], [13] were resistant to AF [14]. The importance of ERα expression in conferring sensitivity of breast cancer cells to AF was further corroborated by evidence that stable transfection of ERα into mesenchymal-like TNBC MDA-MB-231 cells rendered the cells sensitive to AF [15], whereas transient knockdown of ERα in luminal-like breast cancer MCF-7 cells conferred resistance to AF. Combined with the notion that AhR-mediated transcriptional induction of *CYP1A1* is essential for the cytotoxicity of AF, these data not only indicate crosstalk between ERα and AhR pathways in the response of breast cancer cells to AF, but also raise the possibility that reactivation of ERα in mesenchymal-like TNBC cells could restore AhR responsiveness and thus sensitize these cells to AF. There is mounting evidence that histone deacetylase (HDAC) inhibitors, such as vorinostat (also known as SAHA and Zolinza®), given alone or in combination with DNA methyltrasferase (DNMT) inhibitors, restore ERα expression and sensitize ER-negative breast cancers to hormone therapy or chemotherapy [16], [17]. In the present study, we conducted *in vitro* and *in vivo* experiments to examine the combined antitumor effect of vorinostat and AFP464 for treating mesenchymal-like TNBC, and we investigated the underlying molecular mechanisms of that effect.

## Materials and Methods

### Chemicals and Cell Lines

AFP464 and vorinostat were provided by the National Cancer Institute (NCI) Cancer Therapy Evaluation Program (Bethesda, MD). A panel of human breast cancer cell lines (listed in Table 1) as well as MDA-MB-231 cell lines with stable transfection of wild-type ERα (MDA-MB-231/wtERα), mutant ERα (MDA-MB-231/mutERα), or an empty transfection vector (MDA-MB-231/vector) were obtained from the NCI Developmental Therapeutics Program (Bethesda, MD). The human breast cancer cell lines SUM149, SUM 225, and SUM44 were provided by Dr. Stephen Ethier (Karmanos Cancer Institute, Detroit, MI) [18], [19]. The normal breast cell line MCF10A was provided by Dr. Fred Miller (Karmanos Cancer Institute, Detroit, MI) [20]. The NCI cell lines were maintained in RPMI 1640 (Invitrogen, Carlsbad, CA) supplemented with 10% fetal bovine serum (FBS) (Atlanta Biologicals, Lawrenceville, GA), 100 units/ml penicillin, and 100 µg/ml streptomycin (Invitrogen, Carlsbad, CA). The MDA-MB-231/wtERα, MDA-MB-231/mutERα, and MDA-MB-231/vector cell lines were maintained in RPMI 1640 supplemented with 10% FBS and 1 mg/ml G418 (Invitrogen, Carlsbad, CA). SUM149 and SUM225 cell lines were grown in Ham’s F-12 medium supplemented with 5% FBS, 5 µg/ml insulin, and 10 ng/ml epidermal growth factor (EGF). The SUM44 cell line was grown in Ham’s F-12 medium supplemented with 0.5 µg/ml FBS, 5 nM ethanolamine, 10 nM 4-(2-hydroxyethyl)-1piperazineethanesulfonic acid (HEPES), 5 µg/ml transferrin, 10 µM triiodo thyronin, 50 µM selenium, 5 µg/ml insulin, and 1 µg/ml hydrocortisone. The MCF10A cell line was grown in DMEM/F-12 (1∶1, v/v) supplemented with 5% horse serum, 0.029 M sodium bicarbonate, 10 mM HEPES, 10 µg/ml insulin, 20 ng/ml EGF, and 0.5 µg/ml hydrocortisone. All cell lines were maintained at 37°C with 5% CO_2_. The identity of the cell lines was verified by the NCI Developmental Therapeutics Program (http://dtp.nci.nih.gov/branches/btb/characterizationNCI60.html). No authentication was done by us.

**Table 1:** Differential antiproliferative activity of AFP464 in human breast cancer cell lines.

Cell line	Basal/luminal subtype^a^	TNBC subtype^b^	Histology	ER	PR	HER2	P53	BRCA1	AFP464 IC_50_ (µM)^c^
SUM44	Lu		CA	+	−	n/a	wt	wt	0.004
T47D	Lu		IDC	+	+	+	mt	wt	0.014
SKBR3	Lu		AC	−	−	+	mt	wt	0.016
MCF-7	Lu		IDC	+	+	+	wt	wt	0.016
MCF-7/Her2-18	Lu		IDC	+	+	+	wt	wt	0.020
MCF-7/Tam1	Lu		IDC	+	+	+	wt	wt	0.025
HC1937	BaA	BL	IDC	−	−	−	mt	mt	0.010
MDA-MB-468	BaA	BL	AC	−	−	−	mt	wt	0.012
BT20	BaA	Unclassified	IDC	−	−	−	mt	wt	0.020
SUM225	BaA	n/a	IDC	−	−	−	n/a	n/a	0.700
MCF10A	BaB	n/a	Norm.	−	−	−	mt	wt	3
Hs578T	BaB	ML	IDC	−	−	−	mt	wt	20
MDA-MB-231	BaB	ML	AC	−	−	−	mt	wt	25
MX-1	BaB	n/a	IDC	−	−	+	mt	wt	30
SUM149	BaB	ML	Inf.Duc.CA	−	−	−	mt	mt	42

a The luminal/basal subtype obtained from Neve et al [13].

b The TNBC subtype obtained from Lehmann et al [12].

c The cells were treated with AFP464 for 120 h. The IC_50_ value was derived directly from the cell survival curve, shown as the mean value from three independent experiments.

Abbreviations: BaA, basal A; BaB, basal B; BL, basal-like; Lu, luminal; ML, mesenchymal-like; CA, carcinoma; Inf. CA, inflammatory ductal carcinoma; IDC, invasive ductal carcinoma; Norm, normal; ER, estrogen receptor; PR, progesterone receptor; HER2, human epidermal growth factor receptor 2; P53, tumor protein 53; BRCA1, breast cancer type 1 susceptibility protein; MCF-7/Her2-18, herceptin-resistant MCF-7 cell line; MCF-7/Tam1, tamoxifen-resistant MCF-7 cell line; TNBC, triple-negative breast cancer; wt, wild type; mt, mutation; n/a, information not available.

### Transient Knockdown of ERα using siRNA

The siRNA sequence against human ERα (catalog # sc-29305), negative control siRNA-A (sc-37007), and siRNA transfection reagent system (sc-45064) were obtained from Santa Cruz Biotechnology (Santa Cruz, CA). Cell lines were grown in antibiotic-free medium one passage prior to siRNA transfection. The cells were seeded in a 6-well plate, and after 24 h the cells were washed twice with FBS-free and antibiotic-free medium and transfected with ERα siRNA or negative control siRNA according to the manufacturer’s instructions. Briefly, siRNA:transfection reagent (1∶8) mixture complexes were incubated at room temperature for 30 minutes and then added to the ∼80% confluent cells. After incubation for 6–24 h, the complexes were replaced with complete medium, and cells were treated and assayed as indicated below. The knockdown of ERα was confirmed by western blot.

### Cell Proliferation Assay

The antiproliferative activities of AFP464 were evaluated by MTT assay [21] in a panel of cell lines representing normal human mammary epithelial cells (MCF10A) as well as luminal- and basal-like human breast cancer cells (Table 1). Briefly, the subconfluent cells were seeded in 96-well plates, and 24 h after seeding the cells were incubated in culture medium containing AFP464 at concentrations of 0 (control), 0.001, 0.01, 0.1, 0.5, 1, 5, 10, 50, and 100 µM for 120 h. At the end of treatment, cell viability was measured by determining the conversion of MTT to purple formazan by viable cells using a Synergy 2 Plate Reader (550 nm) and Gem51.05 software (BioTEK, Winooski, VT, USA). For each cell line, the MTT assay was performed in 4 replicates in three independent experiments. The concentration that inhibits cell growth by 50% of the control (IC_50_) was obtained by visual inspection of the cell survival-AFP concentration curve.

To assess the role of ERα in the response of breast cancer cells to AFP464, the antiproliferative activity of AFP464 was evaluated in MDA-MB-231 cells stably transfected with wild-type ERα (i.e., MDA-MB-231/wtERα) or mutant ERα (i.e., MDA-MB-231/mutERα) as well as in MCF-7 cells with transient knockdown of ERα. The MDA-MB-231 cells stably transfected with empty transfection vector (MDA-MB-231/vector) and MCF-7 cells transiently transfected with stealth RNAi negative control duplexes were used as controls, respectively. After 24-h incubation with the siRNA transfection complexes, the MCF-7 cells were recovered in fresh complete medium for 2 h and then seeded in 96-well plates. Twenty-four hours after seeding, the cells were treated with AFP464 for 120 h, and cell viability was assessed by MTT assay, as described above.

To further distinguish whether liganded active ERα or expressed unliganded ERα protein mediated the cellular sensitivity to AFP464, the following two experiments were performed. First, the antiproliferative activity of AFP464 was assessed in MCF-7, MDA-MB-231/wtERα, MDA-MB-231/mutERα, and MDA-MB-231/vector cell lines, which were cultured in normal complete medium with 10% FBS or in medium with charcoal-stripped FBS, in the absence or presence of an ERα agonist, 17β-estradiol. In brief, cells were grown in normal complete medium or phenol red-free medium supplemented with charcoal-stripped FBS for 4 passages. Then, the cells were treated with AFP464 for 120 h in the absence or presence of 17β-estradiol (10 nM), and cell viability was assessed by MTT assay. Second, the antiproliferative activity of AFP464 was assessed in the above cell lines, which were cultured in normal complete medium, in the absence or presence of endoxifen. In brief, cells were grown in normal complete medium and treated with AFP464 for 120 h in the absence or presence of endoxifen (50 nM), and cell viability was assessed by MTT assay.

The combined effects of AFP464 and vorinostat on proliferation of MDA-MB-231 and Hs578T cells were assessed by the Chou-Talalay method [22]. The cells were treated with AFP464 and vorinostat, each alone or in combination sequentially (i.e., pretreatment with vorinostat for 24 or 48 h followed by AFP464 for a total of 120 h, or pretreatment with AFP464 for 24 h followed by vorinostat for a total of 120 h) or simultaneously (i.e., co-treatment with vorinostat and AFP 464 for 120 h). For the co-treatment, AFP464 and vorinostat were combined at a fixed concentration ratio of 5∶1 for both cell lines. The treatment (or incubation) time for each drug in the single-agent treatment and in the sequential treatment groups were corresponding and identical (120 h), so that the dose-effect parameters estimated by the Chou-Talalay method could be compared without any bias [22]. For example, for the sequential treatment, MDA-MB-231 cells were treated with vorinostat for 48 h followed by treatment with AFP464 for 72 h. Accordingly, for the vorinostat-only treatment, the cells were treated with vorinostat for 48 h followed by incubation in drug-free medium for 72 h; for AFP464-only treatment, the cells were incubated in drug-free medium for 48 h followed by treatment with AFP464 for 72 h. The combination index (CI) was calculated based on the mean cell proliferation data of two independent experiments using Calcusyn software (Biosoft) [22]. A CI >1.0 indicates an antagonistic effect; CI = 1.0, additive effect; and CI <1.0, synergistic effect.

### Western Blot Analysis of ERα and AhR

Constitutive expression of ERα and AhR in AF-sensitive (i.e., MCF-7, SUM44, MDA-MB-468, and BT20) and AF-resistant (i.e., MDA-MB-231 and Hs578T) human breast cancer cell lines were determined by western blot analysis of whole cell lysates prepared from untreated cells. To determine whether vorinostat could induce ERα and/or AhR protein expression in AF-resistant mesenchymal-like TNBC cells, MDA-MB-231 and Hs578T cells were grown in phenol red-free medium supplemented with charcoal-stripped FBS for 1 week and then treated with vorinostat at their respective IC_50_ values (2.5 or 8 µM) for 6, 12, or 24 h, after which the cells were washed twice with drug-free medium and incubated in fresh medium containing 100 nM 17β-estradiol for 24 h before the cell pellet was harvested and subjected to western blot analysis. Incubation of the cells with 17β-estradiol after vorinostat treatment significantly enhanced the western blot detection signal (shown by our preliminary experiments), probably because the 17β-estradiol-induced homodimerization of ERα could enhance and/or expose antibody-epitope interactions.

Whole cell lysates were prepared from the control and drug-treated frozen cell pellets or snap-frozen fresh tumor tissues, and protein concentration was determined by Bradford assay (Sigma, St. Louis, MO). Lysates were subjected to sodium dodecyl sulfate-polyacrylamine gel electrophoresis and transferred to polyvinylidene difluoride membranes. To detect protein expression of ERα and AhR, membranes were probed with rabbit anti-human ERα polyclonal antibody (1∶1000) (Santa Cruz Biotechnology) and rabbit anti-human AhR antibody (ENZO Life Sciences), respectively, and detected by enhanced chemiluminescence detection reagents (Fisher Scientific, Waltham, MA) using horseradish peroxidase-conjugated goat anti-rabbit IgG (1∶1000) (Santa Cruz Biotechnology) as the secondary antibody. β-actin was used as a loading control.

### Real-time Quantitative Reverse Transcription Polymerase Chain Reaction (qRT-PCR)

To determine whether vorinostat could induce *ERα* transcription and restore AhR responsiveness (as measured by induction of *CYP1A1* and/or *SULT1A1*) in AF-resistant mesenchymal-like TNBC cell lines, qRT-PCR was performed to determine the relative mRNA levels of *ERα*, *CYP1A1*, and *SULT1A1* in MDA-MB-231 and Hs578T cells that were treated with vorinostat (2.5 and 8 µM, respectively) and/or AFP464 (25 and 20 µM, respectively) at their respective IC_50_ values, alone or in sequential combination (i.e., pretreatment with vorinostat for 6, 12, or 24 h followed by 24-h AFP464 treatment).

In addition, to determine whether the loss of ERα activation disrupts AhR responsiveness to AF, ERα was knocked down in vorinostat-pretreated cells using siRNA, and after AFP464 treatment the transcriptional induction of *CYP1A1* and *SULT1A1* was assessed by qRT-PCR. Briefly, MDA-MB-231 and Hs578T cells were pretreated with vorinostat at their respective IC_50_ values (2.5 or 8 µM) for 24 and 12 h, respectively, and then the cells were incubated with the ERα siRNA:transfection reagent (1∶8) mixture complexes in fresh drug-free medium for 4 h. After that, the cells were recovered in fresh complete medium for 2 h and then treated with AFP464 at their respective IC_50_ values (25 or 20 µM) for 24 h.

Total RNA was extracted from the control and drug-treated cell pellets using RNeasy Plus Mini Kit (Qiagen, Valencia, CA). The first-strand cDNA was synthesized using qScript cDNA SuperMix Kit (Quanta Biosciences, Gaithersburg, MD). Real-time PCR was performed using FastStart SYBR Green Master (Roche Applied Science, Indianapolis, IN) on an iCycler iQs real-time PCR detection system (Bio-Rad, Hercules, CA). Thermal cycling conditions for ERα, CYP1A1, and SULT1A1 were: initial denaturing at 95°C for 8 minutes, followed by 40 cycles of denaturing at 95°C for 20 seconds, and combined annealing and extension at 60°C for 20 seconds and 72°C for 20 seconds. Primer sequences used were: human ERα, 5′-CATGCGCTGCGTCGCCTCTAA-3′ (sense), 5′-GCCGCGGCGTTGAACTCGTA-3′ (anti-sense); human CYP1A1, 5′-GCTACCTACCCAACCCTTCC-3′ (sense), 5′-CTCCTTGACCATCTTCTGC-3′ (anti-sense); human SULT1A1, 5′-AGGAGTTCATGGACCACAGC-3′ (sense), 5′-TGAAGGTGGTCTTCCAGTCC-3′ (anti-sense); and human GAPDH, 5′-CTCTCTGCTCCTCCTGTTCGAC-3′ (sense), 5′-TGAGCGATGTGGCTCGGCT-3′ (anti-sense). Gene expression was analyzed using iQ5 Optical system software (Bio-Rad, Hercules, CA). mRNA levels of CYP1A1 or SULT1A1 were normalized to the GAPDH internal standard.

### Immunofluorescence Staining

Indirect immunofluorescence staining was used to examine the cellular localization of AhR, ERα, or γ-H2AX foci formation in the control and drug-treated cells. Cells were grown in 8-well chamber slides under standard cell culture conditions. After a specific treatment, as described above in the section on western blot analysis, the cells were washed with PBS and fixed with ice-cold methanol/acetone solution (1∶1). The slides were rehydrated in PBS for 10 minutes and blocked with 5% bovine serum albumin in PBS at room temperature for 1 hour. Then the slides were incubated with the primary antibodies of anti-human ERα (1∶100) (Invitrogen), AhR (1∶100) (ENZO Life Science), or γ-H2AX (1∶100) (Millipore) overnight at 4°C and incubated with the secondary antibodies TRITC (for positive staining of AhR) or FITC (for positive staining of ERα) at a dilution of 1∶200 at room temperature for 2 h. DAPI (1∶5000 in PBS) was used to stain the nuclei for 2 minutes. Slides were coverslipped with Vectashield antifading reagent (Vecta Laboratories, Burlingame, CA), and images were captured and analyzed using a Leica CTR5500 microscope and OpenLab 5.5.1 software.

### Immunohistochemical Staining

Immunohistochemical staining for human ERα and AhR was performed on tumor sections obtained from MDA-MB-231 xenograft tumor tissues. Briefly, paraffin-embedded tumor sections were de-waxed using a xylene-ethanol series. Endogenous peroxides were removed by incubating the slides in methanol/1.2% hydrogen peroxide. Sections were antigen-retrieved in citrate buffer (pH 6.0) at 95°C for 20 minutes. Then sections were blocked with 10% goat serum in PBS at room temperature for 1 hour, incubated with anti-human AhR antibody (ENZO Life Sciences) (1∶100) or anti-human ERα antibody (1∶100) (Invitrogen) at 4°C overnight, and then incubated with a horseradish peroxidase-conjugated secondary antibody at room temperature for 2 h. Isotype IgGs (Santa Cruz Biotechnology) were used instead of the primary antibody as the negative control. Envision Plus (Dako) was used to develop the signal. Stable diaminobenzidine (Invitrogen) was added for 10 minutes followed by a 2-minute incubation with hematoxylin. The slides were then coverslipped using Eukitt mounting medium (Sigma), and results were documented with a Leica DM5500 microscope. The cytoplasmic and nucleic staining was scored as 0 (negative), 1 (weakly positive), or 2 (strongly positive).

### Animal Studies

To determine the combined antitumor effect of vorinostat and AFP464 *in vivo*, the antitumor activity of vorinostat and AFP464, each given alone or in combination, was evaluated using a mouse xenograft model of basal B subtype (or mesenchymal-like TNBC) MDA-MB-231 cells. In addition, the antitumor activity of AFP464 alone was assessed using a mouse xenograft model of basal A subtype (or basal-like TNBC) MDA-MB-468 cells, which has shown *in vitro* sensitivity to AFP464 and served as a positive experimental control. The animal study was carried out in strict accordance with the recommendations in the National Institutes of Health Guide for the Care and Use of Laboratory Animals. The protocol was approved by the Wayne State University Institutional Animal Care and Use Committee (protocol # A03-10-08).

Female athymic BALB/c mice (5–6 weeks of age) were obtained from NCI Frederick Animal Production Program (Charles River Laboratories, Frederick, MD) and housed under specific-pathogen-free conditions with water and food provided *ad libitum*. The mice were acclimated for 1 week prior to tumor cell implantation. MDA-MB-231 or MDA-MB-468 tumor fragments (30–50 mg) were implanted subcutaneously by trocar in the right and left flank area of each mouse. When established tumors were palpable (i.e., ∼ 10 or 20 days after implantation of MDA-MB-231 or MDA-MB-468 cells, respectively), the mice were randomly assigned to experimental and control groups, and the treatments were initiated.

For the MDA-MB-231 xenograft model, the mice were randomized into 6 groups (7 mice per group). In the combined treatment group, the mice were pretreated with vorinostat (suspended in methylcellulose/0.1% Tween 80 solution, 50 mg/kg) by oral gavage (p.o.) daily for 3 days (i.e., on treatment days −3 to −1 and days 12 to 14) before being treated with AFP464 (dissolved in 5% glucose olution, 35 mg/kg) via tail vein injection (i.v.) on treatment days 1, 3, and 5 of a 14-day cycle for a total of 2 cycles. Accordingly, in the AFP464-only treatment group, the mice were given the vehicle (methylcellulose/0.1% Tween 80 solution) orally for 3 days before being treated with AFP464 at a dose of 35 or 70 mg/kg i.v. on treatment days 1, 3, and 5 of a 14-day cycle for a total of 2 cycles. In the vorinostat-only treatment group, the mice were treated with vorinostat (50 mg/kg) p.o. on days −3 to −1 and days 12 to 14 and given the vehicle (5% glucose olution) at the same time as AFP464 administration in the combined treatment group. In the vehicle control group, the mice were given the vehicle (methylcellulose/0.1% Tween 80 solution or 5% glucose solution) on a schedule matching that of the combined treatment group.

For the MDA-MB-468 xenograft model, the mice were randomly assigned to 3 groups (7 mice per group). For the treatment groups, the mice were treated with AFP464 alone i.v. at a dose of 35 or 50 mg/kg, on days 1, 3, and 5 of a 14-day cycle for a total of 4 cycles. In the control group, the mice were treated with 5% glucose solution i.v. on a schedule matching that of the treatment groups.

Tumor size was measured two or three times per week with a digital caliper. The tumor volume was calculated as 0.5×length×width^2^. Tumor growth inhibition at an indicated time point was expressed as (1−V_T_/V_C_)×100%, where V_T_ and V_C_ are the median tumor volume in the treatment and control groups, respectively. Overall drug tolerance for each treatment was evaluated by body weight changes and general health of the mice throughout the experiments. Body weight was measured daily for the duration of the study. The maximum tolerated dose (MTD) was defined as the dose inducing a maximum loss of body weight of less than 15% and/or no more than 10% treatment-related deaths [23]. When the control group reached humane tumor burden limits (median tumor volume >1000 mm^3^), all mice were euthanized by cervical dislocation, and tumors were surgically removed. Half of the tumor was snap-frozen and used for subsequent western blot analysis of ERα, and the other half was fixed in 10% formalin and embedded in paraffin. Sections (4 µm thick) of tumors were cut and fixed on slides and used for subsequent immunohistochemical staining for ERα and AhR.

### Statistical Analysis

Statistical analyses were performed with SPSS (Version 10.0, SPSS Inc, Chicago, IL). All *P* values were based on two-sided statistical tests, and *P*<0.05 was regarded as statistically significant. The Skewness-Kurtosis All Test indicated that the tumor volume data did not show normal distribution; therefore, a nonparametric Kruskal-Wallis one-way analysis of variance was used to compare the median values of tumor size among the control and drug treatment groups for each xenograft model. Post-hoc paired comparisons after the Kruskal-Wallis test were performed manually using the method described at http://privatewww.essex.ac.uk/~scholp/kw_posthoc.htm.

## Results

### Response of Breast Cancer Cell Lines to AFP464 is Associated with Transcriptional Gene Expression Profiles

The *in vitro* anti-proliferative activity of AFP464 was assessed using a panel of molecularly well-defined human breast cancer cell lines that represent the biological heterogeneity of primary breast cancer (Table 1). According to the distinctive gene clusters of the transcription profiles identified by Neve et al [13], these cell lines are classified as luminal, basal A, and basal B subtypes (Table 1). Luminal-like cell lines are mainly ER-positive and preferentially express genes associated with a more differentiated, noninvasive phenotype [13]. Basal A cell lines display epithelial characteristics and are associated with *BRCA1* gene signatures, whereas basal B cell lines are more aggressive and exhibit mesenchymal and stem cell/progenitor cell-like characteristics [13]. Likewise, recent data from gene expression profiling and clustering analyses reported by Lehmann et al indicate that TNBC cell lines can be classified into 3 main groups: basal-like, mesenchymal-like, and luminal androgen receptor subtype [12]. Lehmann’s gene expression analyses showed that most basal A cell lines belong to the basal-like TNBC subtype, whereas most basal B cell lines fall into the mesenchymal-like TNBC subtype [12]. AFP464 exhibits differential *in vitro* cytotoxic activity in breast cancer cell lines, with an IC_50_ ranging from 0.001–42 µmol/L (Table 1). The response of breast cancer cell lines to AFP464 was associated with transcriptional gene expression profiles: the luminal subtype exhibited the highest sensitivity (IC_50_, <0.001–0.025 µM), followed by the basal A subtype (or basal-like TNBC) (IC_50_, 0.01–0.7 µM), whereas the basal B subtype (or mesenchymal-like TNBC) was resistant (IC_50_≥20 µM) (Table 1).

### ERα Expression is Critical in Mediating Response of Breast Cancer Cells to AFP464

ER-positive breast cancer cell lines, irrespective of resistance to anti-hormone therapies (e.g., tamoxifen-refractory MCF-7/TAM1 and herceptin-refractory MCF-7/Her2-18 cell lines), were sensitive to AFP464, with IC_50_ values ≤0.025 µM, whereas ER-negative breast cancer cell lines with the basal B subtype (e.g., MDA-MB-231 and Hs578T) were resistant to AFP464 (IC_50_ values ≥20 µM) (Table 1). To further prove the importance of ERα expression in the sensitivity of breast cancer cells to AFP464, MDA-MB-231 was stably transfected with human ERα, rendering the cells ER positive. MDA-MB-231/wtERα cell line (IC_50_, 2.5 µM) was ∼ 10-fold and 6-fold more sensitive to AFP464, respectively, than the parental (IC_50_, 25 µM) and empty vector-transfected (IC_50_, 15 µM) cell lines; whereas MDA-MB-231/mutERα cell line was resistant to AFP464 (with a similar IC_50_ as MDA-MB-231/vector), suggesting expression of wild-type but not mutant ERα conferred cellular sensitivity to AFP464. Immunofluorescence staining showed AFP464-induced DNA damage, as assessed by the formation of nuclear γ-H2AX foci (a biomarker for DNA double-strand breaks), in the MDA-MB-231/wtERα cells but not in the MDA-MB-231/vector or parental cell lines when cells were treated with AFP464 at their respective IC_50_ values for 24 h. Moreover, transient knockdown of ERα in luminal-like breast cancer MCF-7 cells rendered the cells ∼20-fold less sensitive to AFP464 compared to siRNA-negative control cells (IC_50_, 0.20 versus 0.01 µM). Collectively, these data indicate a positive role for ERα expression in the response of breast cancer cells to AF or AFP464.

We further experimentally distinguished whether liganded active ERα or unliganded ERα expression mediated the response of breast cancer cells to AFP464. We reasoned that unliganded ERα expression was critical in conferring cellular sensitivity to AFP464, based on the following observations (Table 2): 1) MCF-7 or MDA-MB-231/wtERα cells showed similar sensitivity to AFP464 when cells were cultured in normal complete culture medium with 10% FBS or in medium with charcoal-stripped FBS; 2) MCF-7 or MDA-MB-231/wtERα cells showed similar sensitivity to AFP464 when cells were cultured in normal complete medium in the absence or presence of an ERα agonist, 17β-estradiol (10 nM); and 3) MCF-7 or MDA-MB-231/wtERα cells showed similar sensitivity to AFP464 when cells were cultured in medium with charcoal-stripped FBS in the absence or presence of 17β-estradiol (10 nM). These results collectively suggest that the sensitivity of breast cancer cells, with either endogenous ERα expression (i.e., MCF-7) or transfection of ERα (i.e., MDA-MB-231/wtERα), to AFP464 remains unaltered regardless whether ERα is liganded or not.

**Table 2:** Sensitivity of MCF-7 and MDA-MB-231 (with stable transfection of vector, wild type ERα, or mutant ERα) cell lines to AFP464 in the absence or presence of 17β-estradiol (E2) or endoxifen^a^.

Cell line	Normal medium with 10% FBS		Medium with charcoal-stripped FBS		Normal medium with 10% FBS	
	− E2	+ E2	− E2	+ E2	− Endoxifen	+ Endoxifen
MCF-7	0.020±0.004	0.035±0.006	0.028±0.006	0.035±0.006	0.018±0.004	0.050±0.009*
MDA231/vector	15.5±3.3	16.0±3.7	15.8±3.3	14.5±3.2	ND	ND
MDA231/wtERα	3.0±0.6	3.8±0.8	3.5±0.7	4.0±0.9	3.2±0.6	7.0±1.3*
MDA231/mutERα	15.2±3.4	14.8±3.8	13.4±2.8	12.8±3.0	ND	ND

a Cells were cultured in normal medium with 10% FBS or in medium with charcoal-striped FBS and were treated with AFP464 for 120 h in the absence or presence of 17β-estradiol (E2) (10 nM) or endoxifen (50 nM). Data are expressed as the mean ± standard deviation of the IC_50_ values determined from three independent experiments.

* Statistically significantly different from that in the absence of endoxifen, t-test, *P*<0.05.

ND, not determined.

Interestingly, the presence of endoxifen (50 nM) in normal complete medium reduced the sensitivity of MCF-7 or MDA-MB-231/wtERα cells to AFP464 (Table 2). Endoxifen, a tamoxifen metabolite, is a potent antiestrogen that functions, in a concentration-dependent manner, by targeting ERα for degradation by the proteasome, blocking ERα-mediated transcriptional activation, and inhibiting estrogen-induced breast cancer cell proliferation [24]. It has been reported that endoxifen reduces ERα protein expression in breast cancer cell lines (e.g., MCF-7, T47D, and Hs578t/ERα) at the concentrations of 10 to 1000 nM, whereas it blocks ERα-mediated transcriptional activation and inhibits estrogen-induced breast cancer cell proliferation at higher concentrations (≥100 nM) [24]. We found that treatment with 50 nM of endoxifen for 24 h or 120 h reduced ERα protein expression in MCF-7 or MDA-MB-231/wtERα, but it did not show apparent antiproliferative effect (data not shown here). Therefore, the observed reduced sensitivity of MCF-7 or MDA-MB-231/wtERα to AFP464 in the presence of endoxifen (50 nM) could be attributable to endoxifen-induced ERα degradation. These data further support the notion that ERα expression is critical in mediating the response of breast cancer cells to AFP464.

### AhR Localization in the Cytoplasm is Associated with Cellular Sensitivity to AFP464

**Figure 1:**
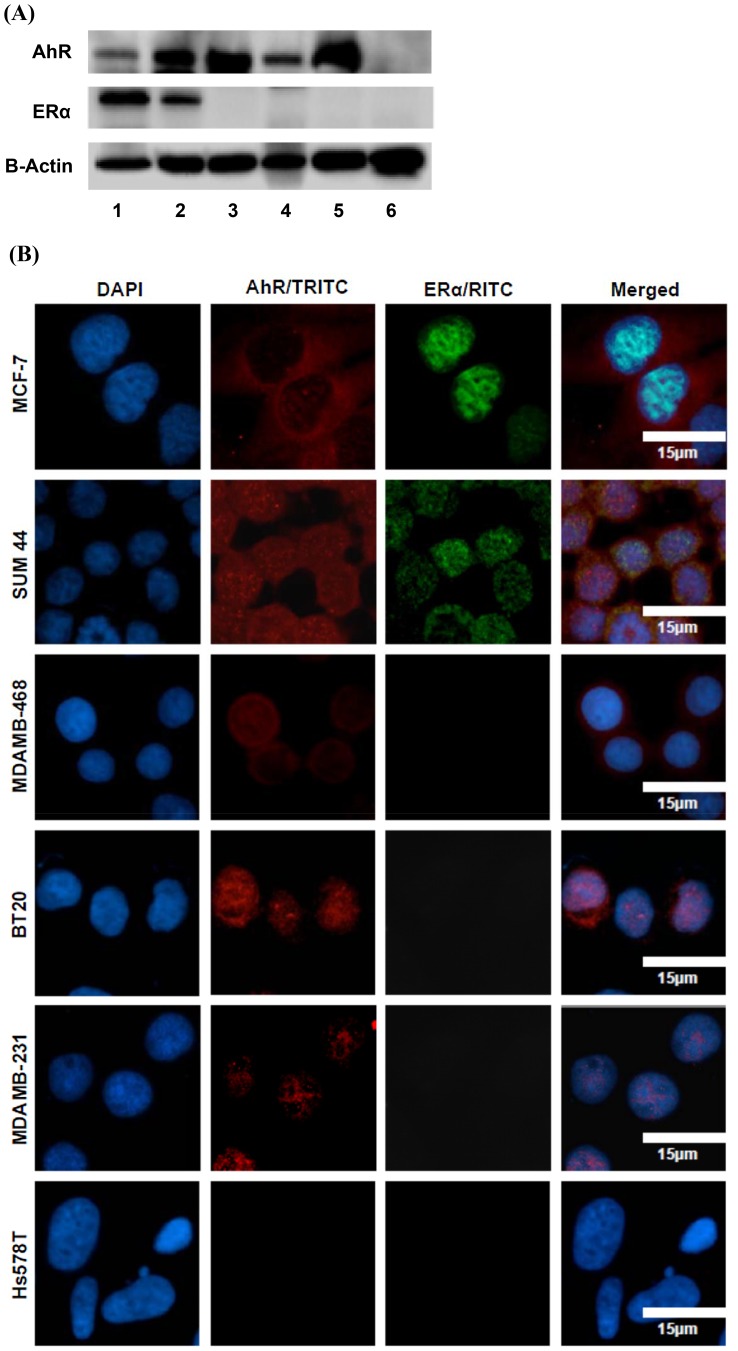
Cytoplasmic localization of the AhR was associated with the sensitivity of breast cancer cell lines to AFP464. (**A**) Western blot shows constitutive expression of ERα and AhR in AFP464-sensitive (MCF-7, SUM44, MDA-MB-468, and BT20) and -resistant (MDA-MB-231, and Hs578T) human breast cancer cell lines (Lanes 1–6, respectively). (**B**) Immunofluorescence staining of the cellular localization of ERα and AhR in AFP464-sensitive and -resistant human breast cancer cell lines. The blue DAPI, red TRITC, and green FITC staining indicates positive staining for the nucleus, AhR, and ERα, respectively.

**Figure 2:**
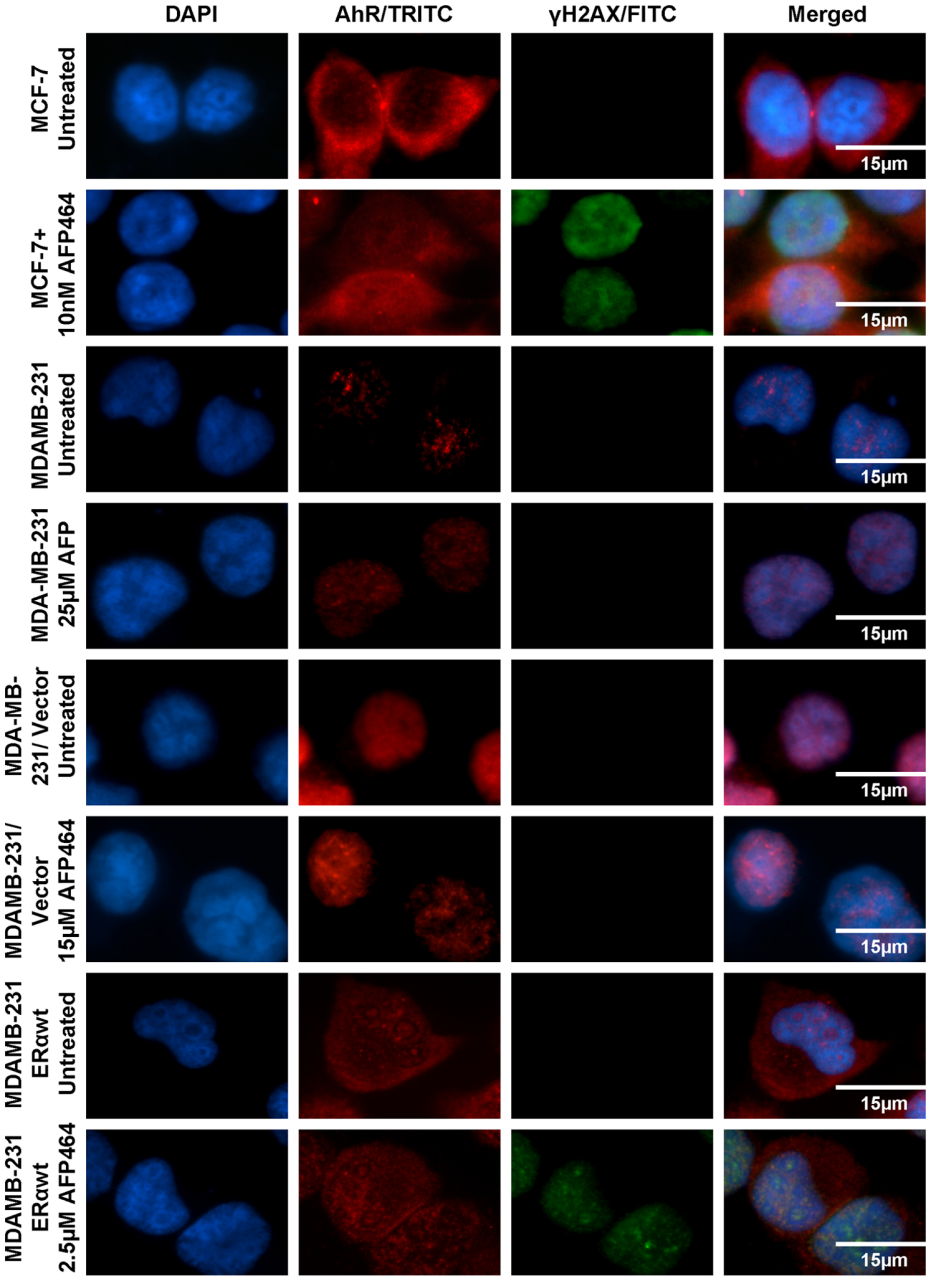
Immunofluorescence staining of AhR and γ-H2AX (a biomarker for DNA double-strand breaks) showed that stable transfection of ERα to MDA-MB-231 cells resulted in translocalization of the AhR from the nucleus to the cytoplasm and rendered cells sensitive to AFP464. The AF-sensitive, ERα-positive MCF-7 cell line was used as a positive control. The AhR was localized predominantly in the cytoplasm in MCF-7 and MDA-MB-231/wtERα cells, but was localized in the nucleus in the parental or empty vector-transfected MDA-MB-231 cells. Nuclear γ-H2AX foci formed in MDA-MB-231/wtERα cells but not in the parental or empty vector-transfected cells when the cell lines were treated with AFP464 at their respective IC_50_ values for 24 h. The blue DAPI, red TRITC, and green FITC staining indicates positive staining for the nucleus, AhR, and γ-H2AX foci, respectively.

The constitutive expression and cellular localization of AhR were assessed by western blot analyses and immunofluorescence staining in 6 human breast cancer cell lines representing AFP464-sensitive (MCF-7, SUM44, MDA-MB-468, and BT20) and -resistant (Hs578T and MDA-MB-231) cell lines. Notably, the AhR was expressed in both cytoplasm and nucleus in AFP464-sensitive ERα-positive luminal subtype breast cancer cell lines (MCF-7 and SUM44), and it was predominantly expressed in the cytoplasm in AFP464-sensitive ERα-negative basal A subtype breast cancer cell lines (MDA-MB-468 and BT20) (Figure 1). By contrast, in AFP464-resistant ERα-negative basal B subtype breast cancer cell lines (Hs578T and MDA-MB-231), AhR was either undetectable at the protein level (e.g., Hs578T) or predominantly localized in the nucleus (e.g., MDA-MB-231) (Figure 1A and 1B). These results suggest that AFP464-sensitive breast cancer cell lines, regardless of ER status, have constitutive cytoplasmic expression of AhR, whereas AFP464-resistant cell lines have undetectable or predominantly nuclear AhR protein expression. This observation agrees with previous studies demonstrating that an AhR-mutated variant of the MCF-7 cell line (AH^R10^), which exhibits constitutive nuclear localization of AhR and expresses low levels of AhR, exhibits cellular resistance to AF and is refractory to CYP1A1 mRNA induction by the drug [4]. We also found that stable transfection of ERα into MDA-MB-231 cells induced translocation of the AhR from the nucleus to the cytoplasm (Figure 2).

### Vorinostat Sensitizes MDA-MB-231 and Hs578T Cells to AFP464

**Figure 3:**
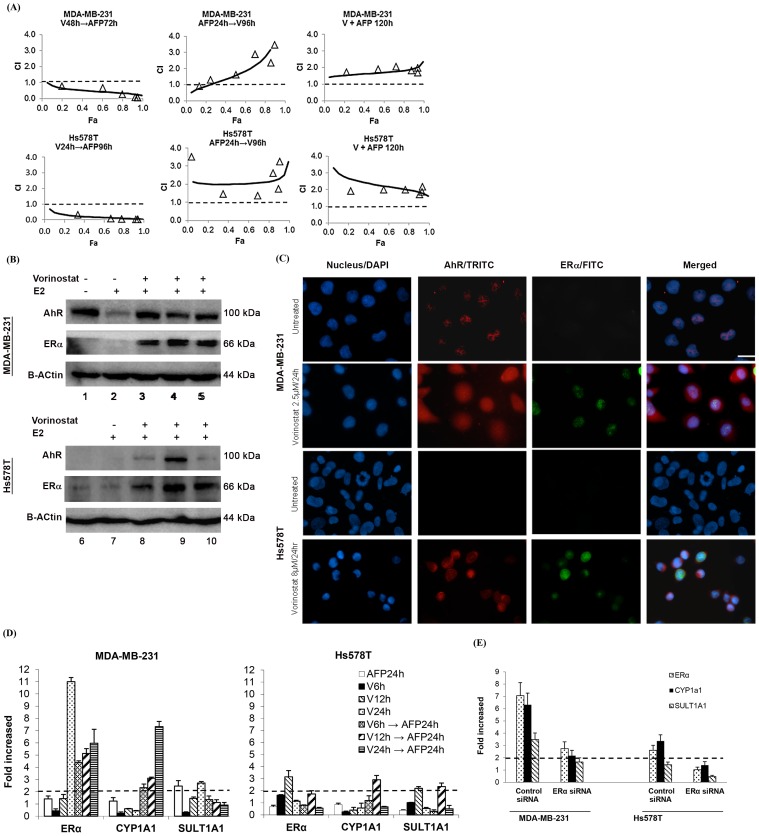
Vorinostat sensitized mesenchymal-like TNBC MDA-MB-231 and Hs578T cells to AFP464 by, at least in part, reactivating ERα expression and restoring AhR responsiveness to AF, as indicated by transcriptional induction of *CYP1A1*. (**A**) Fraction-affected (Fa) versus combination index (CI) plots of sequential or simultaneous treatment with vorinostat (V) and AFP464 (AFP) in MDA-MB-231 and Hs578T cells. AFP464 and vorinostat were combined at a fixed concentration ratio of 5∶1 for both cell lines. The open triangles represent experimental CI values, and the solid lines represent simulated CI values, both of which were calculated by Calcusyn software based on the mean cell proliferation data from two independent experiments. CI >1.0, CI = 1.0, and CI <1.0 indicates antagonistic, additive, and synergistic effects, respectively. (**B**) Western blot of ERα and AhR in control and vorinostat-treated MDA-MB-231 and Hs578T cells. Cells were grown in phenol red-free medium supplemented with charcoal-stripped FBS for 1 week prior to the treatment. MDA-MB-231 and Hs578T cells were treated with vorinostat at their respective IC_50_ values (2.5 and 8 µM) for 6, 12, or 24 h (Lanes 3, 4, and 5, respectively) followed by incubation in fresh medium containing E2 (100 nM) for an additional 24 h. (**C**) Immunofluorescence staining of ERα and AhR in control and vorinostat-treated MDA-MB-231 and Hs578T cells. The cells were treated in the same way as for western blot analysis. The blue DAPI, red TRITC, and green FITC staining indicates positive staining for the nucleus, AhR, and ERα, respectively. (**D**) Real-time RT-PCR determination of relative mRNA levels of *ERα*, *CYP1A1*, and *SULT1A1* in MDA-MB-231 and Hs578T cells treated with vorinostat (V) and AFP464 (AFP) at their respective IC_50_ values, each alone or in sequential combination. (**E**) Real-time RT-PCR demonstrated that transient knockdown of ERα in the vorinostat-pretreated MDA-MB-231 and Hs578T cells diminished AhR-dependent transcriptional induction of *CYP1A1* and *SULT1A1* after AFP464 treatment. MDA-MB-231 and Hs578T cells were pretreated with vorinostat at their respective IC_50_ values (2.5 or 8 µM) for 24 and 12 h, respectively, and then the cells were incubated with the ERα siRNA:transfection reagent (1∶8) mixture complexes in fresh drug-free medium for 4 h. After that, the cells were re-covered in fresh complete medium for 2 h and then treated with AFP464 at their respective IC_50_ values (25 or 20 µM) for 24 h.

The combined antiproliferative effect of vorinostat and AFP464 depends on the sequence of drug administration. Pretreatment with vorinostat followed by AFP464 resulted in a synergistic effect, whereas pretreatment with AFP464 followed by vorinostat or concomitant treatment had no enhanced effect than either drug alone or had an antagonistic effect (Figure 3A). The optimal synergistic effect was observed in MDA-MB-231 cells that were pretreated with vorinostat for 48 h followed by 72 h of AFP464 treatment, with CI values of 0.46, 0.36, and 0.29 at inhibition of 50%, 75%, and 90% of cell proliferation, respectively (Figure 3A). An optimal synergistic effect was observed in Hs578T cells that were pretreated with vorinostat for 24 h followed by 96 h of AFP464 treatment, with CI values of 0.16, 0.09, and 0.06 at inhibition of 50%, 75%, and 90% of cell proliferation, respectively (Figure 3A).

### Vorinostat Reactivates ERα Expression and Restores AhR Responsiveness to AF in TNBC Cells

MDA-MB-231 cells expressed constitutive nuclear AhR, whereas Hs578T cells had undetectable expression (by western blot and immunohistochemistry) of nuclear or cytoplasmic AhR (Figure 3B and 3C). Treatment of MDA-MB-231 and Hs578T cells with vorinostat at their respective IC_50_ values (2.5 and 8 µM) for 6, 12, or 24 h led to reactivation of ERα mRNA expression (using a 2-fold increase in mRNA level as the cut-off value) (Figure 3D) and functional protein (nuclear ERα) expression (Figure 3B and 3C). Furthermore, reactivation of nuclear ERα expression led to translocation of AhR from the nucleus to the cytoplasm in MDA-MB-231 cells, and induced reactivation of cytoplasmic and nuclear AhR expression in Hs578T cells (Figure 3C). Although the exact mechanisms underlying ERα-induced AhR translocation or AhR reexpression remain to be determined, cytoplasmic AhR expression was observed in both MDA-MB-231 and Hs578T cells after vorinostat treatment (Figure 3C).

Consistent with reactivation of ERα protein in vorinostat-treated cells, qRT-PCR analysis demonstrated that vorinostat induced *ERα* mRNA expression (using a 2-fold increase in mRNA level as the cut-off value) in MDA-MB-231 and Hs578T cells, with optimal induction observed at 24 and 12 h of treatment, respectively (Figure 3D). Reactivation of ERα expression was accompanied by restoration of AhR responsiveness to AFP464, as indicated by transcriptional induction of *CYP1A1* (>2-fold increase in mRNA level compared to the untreated control) in cells that were pretreated with vorinostat followed by 24-h AFP464 treatment but not in those treated with either drug alone (Figure 3D). In agreement with the optimal time for induction of ERα by vorinostat, the optimal induction of *CYP1A1* mRNA after AFP464 treatment was observed at 24 and 12 h of pretreatment with vorinostat in MDA-MB-231 and Hs578T cells, respectively (Figure 3D).

To further demonstrate the key role that ERα plays in AhR responsiveness to AF, ERα was transiently knocked down using siRNA in vorinostat-pretreated MDA-MB-231 and Hs578T cells, and the induction of *CYP1A1* and *SULT1A1* after AFP464 treatment was determined by qRT-PCR. We found that the loss of ERα reactivation in vorinostat-pretreated cells diminished induction of *CYP1A1* and *SULT1A1* after AFP464 treatment (Figure 3E).

### Vorinostat Potentiates AFP464 Antitumor Activity in an MDA-MB-231 Xenograft Model

**Figure 4:**
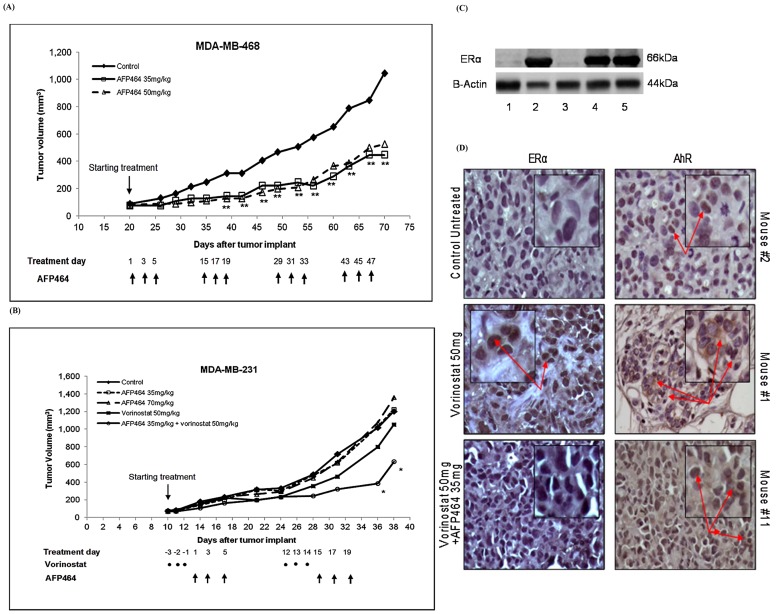
Antitumor activity of AFP464 and vorinostat, alone or in combination, in xenograft models using basal A subtype MDA-MB-468 and basal B subtype (or mesenchymal-like TNBC) MDA-MB-231 cells. (**A**) Tumor growth in the control and drug treatment groups. Data are expressed as the median tumor volume of 10–14 tumors. *Kruskal-Wallis test, the median tumor volume in the combined treatment group was significantly different from those in the control and AFP464-only treatment groups in the MDA-MB-231 xenograft model, *P*<0.05. **Kruskal-Wallis test, the median tumor volume for each AFP464 treatment group was significantly different from that of the control group in the MDA-MB-468 xenograft model, *P*<0.01. (**B**) Western blot of ERα protein expression in the tumor tissues that were collected from the control and vorinostat/AFP464-treated mice. MDA-MB-231 and MDA-MB-231/wtERα cell lines were used as the ERα-negative and -positive controls, respectively. Lanes 1 and 2 were whole cell lysates from MDA-MB-231 and MDA-MB-231/wtERα cell lines, respectively; lanes 3–5 are MDA-MB-231 xenograft tumor tissue lysates obtained from mice treated with the vehicle solution (control), vorinostat 50 mg/kg, or a combination of vorinostat (50 mg/kg) and AFP464 (35 mg/kg), respectively. (**C**) Immunohistochemical staining for AhR and ERα in the tumor tissues that were collected from the same mice as those used for the western blot analysis.

The basal A subtype breast cancer cells, i.e., MDA-MB-468, were sensitive to AFP464 *in vitro*, with an IC_50_ of 0.012 µM (Table 1). AFP464 showed *in vivo* antitumor activity in the MDA-MB-468 xenograft model, as evidenced by statistically significantly delayed tumor growth in mice treated with 35 or 50 mg/kg AFP464 compared to mice treated with vehicle control (Figure 4A). Both dose levels were well tolerated and produced equivalent antitumor activity. After one and two cycles of AFP464 treatment, the median tumor growth was inhibited by 57% and 54%, respectively, compared to the control, *P*<0.01.

In contrast, AFP464 alone did not show antitumor activity (0% inhibition) at a dose of either 35 or 70 mg/kg in the xenograft model using mesenchymal-like TNBC MDA-MB-231 cells (Figure 4B). AFP464 was well tolerated at a dose of 35 mg/kg, but 70 mg/kg induced up to 15% body weight loss and the death of 1 of 7 mice during the course of treatment; therefore 70 mg/kg was defined as the MTD. When combined with vorinostat (50 mg/kg, p.o.), AFP464 was well tolerated at a dose of 35 mg/kg, but at a dose of 70 mg/kg, 50% of the mice in that treatment group died by the end of two cycles of treatment; thus 35 mg/kg was defined as the MTD of AFP464 in combination with vorinostat. The combined treatment with vorinostat (50 mg/kg) and AFP464 (35 mg/kg) inhibited tumor growth compared to the control or treatment with AFP464 alone (*P*<0.05) (Figure 4B), as indicated by 51% inhibition of tumor growth after two cycles of treatment. In the MDA-MB-231 xenograft model, tumor growth inhibition induced by vorinostat plus AFP464 treatment (51%) was comparable to AFP464-only treatment in the AF-sensitive MDA-MB-468 xenograft model (54%). This provides *in vivo* evidence that vorinostat sensitized AF-resistant mesenchymal-like TNBC cells (i.e., MDA-MB-231) to AFP464.

Western blot and immunohistochemical analyses of MDA-MB-231 xenograft tumor tissues showed that ERα expression was activated in tumor tissues collected from mice treated with vorinostat alone or in combination with AFP464, but not in tissues from control untreated mice (Figure 4B and 4C). In addition, immunohistochemical staining suggested that vorinostat treatment induced cytoplasmic expression of AhR in MDA-MB-231 xenograft tumor tissues (Figure 4C). These *in vivo* findings support our hypothesis that vorinostat sensitizes tumors to AFP464 treatment in part by reactivating ERα and restoring the responsiveness of AhR to AF.

## Discussion

Epigenetic events, such as DNA methylation and chromatin remodeling, are involved in regulating gene expression. Chromatin is a dynamic complex composed of DNA, histones, and non-histone proteins. Chromatin remodeling involves enzymatic modifications (such as acetylation and methylation) of histones, and it affects the accessibility of regulatory proteins to DNA, resulting in transcriptional activation or repression. For example, histone acetylation by histone acetyltransferases promotes chromatin expansion, allowing transcriptional activation. Conversely, histone deacetylases (HDACs) catalyze the removal of acetyl groups from lysine residues of core histones (particularly H3 and H4), thereby increasing ionic interactions between positively charged lysines of histones and negatively charged DNA, which results in chromatin condensation and transcriptional repression [25]. Inhibition of HDACs has been shown to be a promising therapeutic intervention to reverse aberrant epigenetic alterations associated with cancer. For example, several novel HDAC inhibitors are currently being evaluated in clinical trials as monotherapy or in combination with other treatments in patients with hematologic or solid tumors. Vorinostat is the first in its class to be approved by the Food and Drug Administration for the treatment of cutaneous T-cell lymphoma [26]. It inhibits the activity of class I and II HDACs, including HDAC1, HDAC2, HDAC3, and HDAC6, at low micromolar concentrations [27]. Vorinostat and other HDAC inhibitors have shown synergistic or additive antitumor effects with a wide range of cancer treatment modalities, including chemotherapy, molecularly targeted therapy, and radiation therapy, by various mechanisms, some unique for particular combinations [28].

Here, we demonstrated that the combination of vorinostat and AFP464 had a synergistic antiproliferative effect, in a schedule-dependent manner, in mesenchymal-like TNBC cell lines (i.e., MDA-MB-231 and Hs578T) and, moreover, that vorinostat potentiated *in vivo* antitumor activity of AFP464 in an MDA-MB-231 xenograft model. We reason that the synergistic antitumor effect of these two drugs is attributed, at least in part, to epigenetic reactivation of ERα expression by vorinostat, which restores AhR responsiveness to AF. This conclusion is based on the following observations: 1) Vorinostat reactivated ERα expression at both transcriptional and protein levels in MDA-MB-231 and Hs578T cells (Figure 3B and 3D). 2) Reactivation of ERα expression was accompanied by restoration of AhR responsiveness to AF, as indicated by transcriptional induction of *CYP1A1*, in MDA-MB-231 and Hs578T cells (Figure 3D), whereas the loss of ERα activation in vorinostat-pretreated cells diminished AhR-dependent transcriptional induction of *CYP1A1* after AF treatment (Figure 3E). 3) Vorinostat-induced restoration of ERα expression and cytoplasmic AhR expression was also observed *in vivo* in MDA-MB-231 xenograft tumor tissue (Figure 4C and 4D).

The observed reactivation of ERα expression by vorinostat in MDA-MB-231 and Hs578T cells is in agreement with previous reports that HDAC inhibitors induce reactivation of the expression of *ERα* mRNA and functional protein in ER-negative breast cancer cell lines through, at least in part, an epigenetic mechanism [17], [25], [29], [30]. It is well known that the epigenetic silencing of the ERα gene in ER-negative human breast cancers involves interactions between DNA methyltrasferases (DNMTs) and histone deacetylases (HDACs) to maintain a stable repressive chromatin complex in the ER promoter. The silenced ERα promoter has been associated with DNA hypermethylation, histone hypoacetylation, histone H3 lysine 9 (H3–K9) methylation, and a transcriptional co-repressor complex containing HDAC1, HDAC2, DNMT1, DNMT3a, DNMT3b, and methyl-CpG binding domain proteins (MeCP2, MBD1, MBD2) [31]–[33]. Epigenetic reactivation of ERα by HDAC inhibitors alone or in combination with DNMT inhibitors involves reorganizing chromatin structure by modifying core histones and modulating the binding of various nonhistone proteins. It has been shown that treatment with HDAC inhibitors (such as LBH489) releases DNMT1, HDAC1, and the H3–K9 methyltransferase SUV39H1 from the ERα promoter, thereby creating an active chromatin structure that manifests as accumulation of acetylated histones H3 and H4, suppression of H3–K9 methylation, and impaired binding of heterochromatin protein 1 at the promoter [29]–[31]. It is likely that vorinostat reactivates ERα expression through a molecular mechanism similar to that of LBH489, as both are hydroxamate HDAC inhibitors.

Strikingly, vorinostat-induced reactivation of ERα expression was accompanied by restoration of AhR-dependent transcriptional induction of *CYP1A1* in MDA-MB-231 and Hs578T cells (Figure 3D). The observed restoration of AhR responsiveness could be attributed, at least in part, to the ERα-AhR crosstalk mechanism. ERα and AhR are both ligand-activated transcription factors that regulate gene expression through two different but not mutually exclusive mechanisms–via direct DNA binding to their response elements or by protein-protein interactions to recruit other transcription factors and coregulators to targeted *cis*-regulatory elements [34]–[38]. Crosstalk between ERα and AhR has been well established based on the findings that ERα and AhR are reciprocally recruited to AhR and ERα *cis*-regulatory elements [36], [39]–[41]. Although ERα–AhR crosstalk exhibits agonist-dependent, cell type- and species-specific characteristics [36], [39]–[41], many studies suggest that ligand-activated AhR inhibits ERα signaling, whereas unliganded or ligand-activated ERα positively modulates AhR-mediated transcription [42], [43]. The positive role of ERα in AhR activity is evidenced by a number of findings. For example, activation of AhR target genes by a typical ligand of AhR, 2,3,7,8-tetrachlorodibenzo-p-dioxin (TCDD), correlated with constitutive ERα expression levels in breast cancer cell lines [44]–[46]. In addition, transfection of ERα into ER-negative breast cancer cell lines restored AhR responsiveness to TCDD as determined by induction of *CYP1A1* [47], [48]. Consistently, we demonstrated that transfection of ERα into MDA-MB-231 cells rendered cells sensitive to AFP464 (Figure 2), which could be explained by the restoration of AhR responsiveness to AF by endogenous introduction of ERα. Furthermore, others demonstrated that stable knockdown of ERα expression by short-hairpin RNA in T47D human breast cancer cells and HC11 mouse mammary cells significantly reduced TCDD-induced *CYP1A1* expression [39], [40]. We demonstrated that transient knockdown of ERα by siRNA in luminal-like breast cancer MCF-7 cells reduced their sensitivity to AFP464 by ∼20-fold. The positive role of ERα in AhR responsiveness is further corroborated by *in vivo* evidence that ERα knockout mice showed decreased AhR-dependent induction of *CYP1A1* mRNA compared to wild-type mice [39]. The precise molecular mechanisms by which ERα modulates AhR-mediated transcription are yet to be fully elucidated. It has been proposed that ERα acts as a co-regulator of AhR-mediated transcriptional activation via protein-protein interaction with the activated AhR/ARNT heterodimer complex in the AhR target gene promoter [39], [40]. In accordance with the notion that ERα is a positive modulator of AhR responsiveness, our *in vitro* and *in vivo* studies reveal that reactivation of ERα expression by vorinostat restores AhR responsiveness to AF (Figures 3 and 4). This provides, at least in part, a molecular basis for the observed synergistic antitumor activity of vorinostat and AFP464 in treating mesenchymal-like TNBC.

Besides epigenetic modulation of ERα, other molecular mechanisms might also contribute to the synergistic antitumor activity of vorinostat and AFP464. The mechanisms of action of HDAC inhibitors are complex, involving both transcription-dependent and transcription-independent factors. HDAC inhibitors selectively alter gene transcription, in part, by chromatin remodeling and by changing the structure of proteins in transcription factor complexes [49]. In addition, HDAC inhibitors can interact with many other non-histone protein substrates (such as DNA binding transcriptional factors, transcription co-regulators, hormone receptors, DNA repair enzymes, chaperone proteins, and cytoskeletal proteins), which are involved in regulating gene expression, cell proliferation, and cell death [50]. Microarray analyses suggest that HDAC inhibitors can alter the transcription of as many as 7–10% of genes (using a 2-fold change as the cut-off value) in cell lines from patients with leukemia, multiple myeloma, and carcinomas of the colon, bladder, kidney, prostate, and breast [51]–[55]. Notably, a recent study showed that treatment of MDA-MB-231 cells with the hydroxamate HDAC inhibitor panobinostat (LBH489) induced upregulation of many anti-proliferative, tumor-suppressor, and epithelial marker genes, and strikingly, it initiated partial reversal of the epithelial-to-mesenchymal transition in MDA-MB-231 cells [56]. These data suggest that HDAC inhibitors can alter the gene expression profile of mesenchymal-like TNBC cells (such as MDA-MB-231) to become less aggressive and to have a more favorable prognostic profile. In agreement with these findings, our preliminary data from microarray analyses using the Illumina Human HT-12 v3 whole-genome expression BeadChips revealed that treatment of MDA-MB-231 cells with vorinostat at its IC_50_ (2.5 µM) for 48 h altered its gene expression profile from the basal B to a luminal-like profile (unpublished data not shown here). This could provide another molecular basis by which vorinostat sensitizes mesenchymal-like TNBC cells to AF or AFP464, given the association between the gene expression profile and the response of breast cancer cells to AF as demonstrated in this study (Table 1). The differential sensitivity of basal-like and mesenchymal-like TNBC cell lines to AF or AFP464 could be attributable to their distinct “driver” signaling pathways. The basal-like TNBC cell lines express high levels of genes involved in cell proliferation as well as DNA damage-response genes, suggesting that patients with basal-like TNBCs would likely benefit from agents that target highly proliferative tumor cells (e.g., anti-mitotic and DNA-damaging agents) [12]. In accordance with this notion, it has been reported that patients with basal-like TNBCs have better clinical response from taxane-based and radiation-based treatment than patients with tumors that display characteristics of the mesenchymal-like subtype [57], [58]. Thus, it was not surprising that basal-like TNBC cell lines were sensitive to AFP464, which exerts its antitumor activity by biotransformation to DNA-damaging species in cancer cells. In contrast, the AF-resistant mesenchymal-like TNBC cell lines have enriched expression of genes associated with the epithelial-to-mesenchymal transition (e.g., TGFβ, mTOR, ALK, Wnt/β-catenin) and cell motility (Rac1/Rho, focal adhesion, integrin signaling) pathways [12], suggesting that drugs targeting these aberrant pathways could be used to treat mesenchymal-like TNBC.

## Conclusions

AF or AFP464 represents a new class of cytotoxic agents with a unique mechanism of action. AF exerts its antitumor activity primarily, if not exclusively, by inducing AhR-dependent transcriptional activation of metabolizing enzymes, including but not limited to CYP1A1, which convert AF to DNA-damaging species. In the present study, we demonstrated that the response of breast cancer cells to AF or AFP464 was associated with their gene expression profile, indicating the usefulness of gene expression profiling in selecting patients for AFP464 treatment. Although treatment with AFP464 alone could be an option for patients with luminal and basal A subtype breast cancers, patients with basal B subtype or mesenchymal-like TNBC might require combined treatment with AFP464 and gene expression-modifying agents, such as HDAC inhibitors. Here, we provide *in vitro* and *in vivo* evidence that the HDAC inhibitor vorinostat sensitizes mesenchymal-like TNBC to AFP464, at least in part, through reactivation of ERα expression and restoration of AhR responsiveness to AF. This study not only provides insights into the molecular mechanisms of action of AF (a ligand of AhR), given alone and in combination with vorinostat (a HDAC inhibitor), but also opens new possibilities for a molecularly targeted approach to treating aggressive mesenchymal-like TNBC.
